# Serum Activin A and Follistatin Levels in Gestational Diabetes and the Association of the Activin A-Follistatin System with Anthropometric Parameters in Offspring

**DOI:** 10.1371/journal.pone.0092175

**Published:** 2014-04-24

**Authors:** Silvia Näf, Xavier Escote, Mónica Ballesteros, Rosa Elena Yañez, Inmaculada Simón-Muela, Pilar Gil, Gerard Albaiges, Joan Vendrell, Ana Megia

**Affiliations:** 1 Endocrinology and Diabetes Unit, Hospital Universitari Joan XXIII, Tarragona, Spain; 2 Obstetrics and Gynecology Service, Hospital Universitari Joan XXIII, Tarragona, Spain, IISPV, Universitat Rovira i Virgili, Tarragona, Spain; 3 Centro de Investigación Biomédica en Red de Diabetes y Enfermedades Metabólicas Asociadas (CIBERDEM), Instituto de Salud Carlos III, Madrid, Spain; 4 Institut d'Investigació Sanitària Pere Virgili (IISPV), Universitat Rovira i Virgili, Tarragona, Spain; Faculty of Medicine & Allied Sciences, Rajarata Univeresity of Sri Lanka, Sri Lanka

## Abstract

**Context:**

The Activin A-Follistatin system has emerged as an important regulator of lipid and glucose metabolism with possible repercussions on fetal growth.

**Objective:**

To analyze circulating activin A, follistatin and follistatin-like-3 (FSTL3) levels and their relationship with glucose metabolism in pregnant women and their influence on fetal growth and neonatal adiposity.

**Design and methods:**

A prospective cohort was studied comprising 207 pregnant women, 129 with normal glucose tolerance (NGT) and 78 with gestational diabetes mellitus (GDM) and their offspring. Activin A, follistatin and FSTL3 levels were measured in maternal serum collected in the early third trimester of pregnancy. Serial fetal ultrasounds were performed during the third trimester to evaluate fetal growth. Neonatal anthropometry was measured to assess neonatal adiposity.

**Results:**

Serum follistatin levels were significantly lower in GDM than in NGT pregnant women (8.21±2.32 ng/mL vs 9.22±3.41, *P* = 0.012) whereas serum FSTL3 and activin A levels were comparable between the two groups. Serum follistatin concentrations were negatively correlated with HOMA-IR and positively with ultrasound growth parameters such as fractional thigh volume estimation in the middle of the third trimester and percent fat mass at birth. Also, in the stepwise multiple linear regression analysis serum follistatin levels were negatively associated with HOMA-IR (β = −0.199, *P* = 0.008) and the diagnosis of gestational diabetes (β = −0.138, *P* = 0.049). Likewise, fractional thigh volume estimation in the middle of third trimester and percent fat mass at birth were positively determined by serum follistatin levels (β = 0.214, *P* = 0.005 and β = 0.231, *P* = 0.002, respectively).

**Conclusions:**

Circulating follistatin levels are reduced in GDM compared with NGT pregnant women and they are positively associated with fetal growth and neonatal adiposity. These data suggest a role of the Activin-Follistatin system in maternal and fetal metabolism during pregnancy.

## Introduction

Gestational diabetes mellitus (GDM) is one of the most frequent metabolic disorders complicating pregnancy. Late pregnancy is a state of physiological insulin resistance in which nutritional, hormonal and inflammatory factors are involved. When pregnant women fail to overcome this insulin resistance, GDM develops [1]. This metabolic disturbance modifies the in utero environment affecting fetal development and favoring fetal overgrowth and adipose tissue accretion. Current treatment of GDM is aimed at normalizing birth weight and reducing fat mass [2]. Obstetric ultrasound allows assessing fetal growth despite the poor correlation reported between estimated fetal weight and neonatal adiposity [3], [4]. Recently it has been shown that the measurement of fractional limb volume, a soft tissue parameter of fetal body composition, is more accurate to monitor fetal nutritional status and is closely related to neonatal adiposity [4].

The Activin A-Follistatin system has emerged as an important regulator of lipid and glucose metabolism with possible implications on fetal growth [5]. Activin A, a member of the transforming growth factor-β (TGF-β) superfamily, is a pleitropic cytokine that regulates several cellular events including a role in glucose homeostasis. Enhanced glucose stimulated insulin secretion [6] and increased beta cell proliferation in cultured rat and human islets by activin A [7], [8] have been reported. Furthermore, it plays a role in lipid homeostasis by promoting proliferation and inhibiting adipocyte differentiation from human preadipocytes [9].

Follistatin and follistatin-like-3 (FSTL3) are high-affinity activin binding proteins, which neutralize most of its biological actions [10]. Regarding adipose tissue, in contrast to activin A, follistatin promotes adipogenic differentiation of progenitor cells [11]. Interestingly, FSTL3 knockout mice develop metabolic alterations including enlarged islets, β-cell hyperplasia, decreased visceral fat mass, improved glucose tolerance and insulin sensitivity [12].

The feto-placental unit is the main source of serum activin A levels during pregnancy [13], [14] and follistatin and FSTL3 are also highly expressed by the placenta [15], [16] and fetal membranes [16]

Data assessing serum activin A levels in pregnancies complicated by GDM are scarce. Higher concentrations of activin A in two small cohorts of pregnant women with GDM have been described [17], [18]. Previous studies regarding FSTL3 circulating levels in GDM women yielded heterogeneous results. Thus, in the first trimester, low [19] or unchanged [20] circulating levels have been reported in women who developed GDM. Likewise in the third trimester, at term, low serum FSTL3 levels have been observed in GDM women [21]. To our knowledge there are no reports concerning follistatin levels in GDM.

Considering the implication of the Activin-Follistatin system in glucose and lipid metabolism, key elements in insulin resistance developed during pregnancy, we hypothesized that serum levels of activin A, follistatin and FSTL3 would be associated with pregnancies affected by GDM. To test this hypothesis, we analyzed circulating activin A, follistatin and FSTL3 levels in a well-characterized cohort of pregnant women with GDM and in their normal glucose tolerance (NGT) counterparts. Furthermore, we also analyzed their relationship with the ultrasound and anthropometric parameters of their offspring.

## Materials and Methods

A prospective case-control study was conducted at the Joan XXIII University Hospital. The study protocol and laboratory methods have been fully described previously [22]. Briefly, pregnant Caucasian women included in this study were recruited at the time of antepartum screening for GDM (between 26–30 weeks). All of the women who participated underwent a 3 hour, 100 g oral glucose tolerance test (OGTT) and were monitored from the time of inclusion until delivery. Baseline serum and plasma samples obtained at the time of the OGTT were kept in a GDM biobank collection at our institution. The Joan XXIII University Hospital Ethics Committee approved the study, and written informed consent was obtained from all participants. Following the Spanish GDM guidelines and according to the OGTT, women with two or more values above the threshold proposed by the National Diabetes Data Group [23], [24] were considered GDM, and women for whom all the values were below the threshold were classified in the normal glucose tolerance (NGT) group. Women with only one value above the threshold after oral glucose tolerance were excluded from the study. Two hundred and seven pregnant women who fulfilled the following criteria at the end of pregnancy were recruited for this study: 1) a singleton pregnancy, 2) accurate gestational age confirmed by an ultrasound examination before 20 weeks of gestation, 3) the absence of fetal abnormalities identified at birth, 4) NGT or GDM diagnosed before 30 weeks of pregnancy. One hundred and ninety-seven women fulfilled an additional criterion: 5) at least two ultrasound explorations, one upon recruitment and another in the middle of the third trimester. GDM women were given a personalized diet with 40% of carbohydrates and they were instructed to self-monitor blood glucose daily 6 times a day (fasting and 1 hour postprandial). Insulin therapy was recommended when fasting glucose values were repeatedly ≥95 mg/dl and/or 1 hour postprandial values were >140 mg/dl. According to these criteria, 49 women were treated only with diet and 29 women required the addition of insulin.

### Clinical and demographic data

Upon inclusion, demographic and historical information was collected via an interviewer-administered questionnaire focused on personal medical and obstetrical history and information regarding the current pregnancy with special attention to risk factors for gestational diabetes. Also, maternal anthropometric data including height, pre-pregnancy weight, and weight at the end of pregnancy were collected. Pre-pregnancy BMI was calculated using the formula: pre-pregnancy weight (kg)/(height (m))^2^. Increased BMI was calculated by the formula BMI gain = final BMI – pre-pregnancy BMI. Neonatal length and weight were determined in all participants using a measuring board to the nearest 0.1 cm and a calibrated scale to the nearest 10 g. Birth weight (BW) was transformed into standard deviation scores (SDS) to adjust for gestational age at delivery using gender-specific references of fetal growth [25]. In a subgroup of one hundred and sixty five offspring, more complete neonatal anthropometric measurements were taken. Triceps, biceps, subscapular, and flank skinfold thickness were measured with Holtain skinfold callipers (Chasmors Ltd, London UK). Percent fat mass (PFM) at birth was estimated by the formula validated by Catalano (fat mass (FM) = 0.39055 (birth weight)+0.0453 (flank skinfold)−0.03237 (length)+0.54657/100 [26].

### Fetal ultrasound

One examiner performed all ultrasound examinations using ultrasound color Doppler equipment (RAB 4–8L, Voluson 730 Expert, General Electric Medical Systems, Austria) using hybrid mechanical and curved array abdominal ultrasonic transducers. Fetal weight estimations (FWE) were calculated at approximately 28 (range, 27–30) (FWE_28_) and 35 (range, 34–36) (FWE_35_) weeks' gestation using Hadlock's equation [27]. Fractional thigh volume estimations (FTVE) were also calculated at approximately 28 (range, 27–30) (FTVE_28_) and 35 (range, 34–36) (FTVE_35_) weeks' gestation according to the method provided by Lee et al. [28]. FWE and FTVE were transformed into standard deviation scores (SDS). To adjust for gestational age, we used gender-specific references of fetal growth in the Spanish population for FWE [25] and charts published by Lee for FTVE [28].

### Laboratory measurements

The 100 g-OGTT was performed in the morning after an overnight fasting. Venous blood samples were drawn at baseline and 60, 120 and 180 minutes after ingesting a standard 100-g glucose load. Serum glucose levels were determined in an ADVIA 2400 (Siemens AG, Munich, Germany) autoanalyzer using the standard enzyme methods. Fasting plasma insulin and C-peptide were determined by immunoassay in an ADVIA Centaur System (Siemens AG, Munich, Germany). This assay shows a cross-reactivity of lower than 0.1% to intact human proinsulin and the primary circulating split form. Homeostasis model of insulin resistance (HOMA-IR) index was determined according to the following equation: fasting plasma glucose (mmol/L)×fasting plasma insulin (µU/ml/22.5) [29].

Also activin A, follistatin and FSTL3 were measured in the serum obtained at the time of the OGTT. Serum activin A levels were measured by sandwich ELISA (R&D Systems Europe Ltd, Abingdon, UK). The intra- and inter-assay CVs were 4.2% and 5.8%, respectively and assay sensitivity was 3.67 pg/ml. Serum follistatin levels were determined using a human ELISA kit (R&D Systems Europe Ltd,). The intra and inter-assay CVs were 2.3% and 8% respectively and assay sensitivity was 29 pg/ml. Serum FSTL3 levels were determined using a human ELISA kit (R&D Systems Europe Ltd). The intra and inter-assay CVs were 2.4% and 5.3%, respectively and assay sensitivity was 3.68 pg/ml.

### Statistical analysis

Statistical analysis was performed by using the SPSS statistical package (version 13; SPSS, Chicago, IL). The 1-sample Kolmogorov-Smirnov test was performed to verify the normal distribution of the quantitative variables. For clinical and anthropometrical variables, normal distributed data are expressed as mean values (±SD), and for variables with a non-Gaussian distribution, values are expressed as median (25^th^–75^th^ percentile). Categorical variables were reported by frequencies (percentages). Comparisons of quantitative variables between groups were performed by either Student's t-test or the Mann-Whitney U test, according to data distribution. Associations between quantitative variables were evaluated by Pearson's correlation analysis. Activin A, pre-pregnancy BMI, insulin and HOMA-IR whith a skewed distribution were log transformed.

The relationship of maternal activin A, follistatin and FSTL3 with clinical and analytical variables was evaluated by stepwise multiple linear regression analysis. Variables with a significant association in the bivariate analysis or those known to be related with the physiopathology of insulin resistance were included in the model as covariates. To avoid multicollinearity we assessed the variable inflation factor (VIF) for all the covariates in each regression model and no variable included had a VIF greater than 2. The main determinants of BW ZS, PFM at birth, FTVE_28_ SDS, FTVE_35_ SDS, FWE_28_ SDS and FWE_35_ SDS were evaluated by stepwise multiple regression analysis. Statistical significance was accepted at the level P<0.05.

The sample size for this study was calculated according to activin A values. In previous literature [17], it has been observed that activin levels in pregnancy were 1.5 units higher in women with GDM than in NGT women. Accepting an alpha risk of 0.05 and a beta risk of 0.2 in a two-sided test we calculated that 128 subjects were necessary in the first group and 76 in the second to recognize as statistically significant a difference greater than or equal to 1.5 units, when a common standard deviation was assumed to be 3.7.

## Results

One hundred and twenty-nine NGT and seventy-eight GDM pregnant women and their respective offspring were included in the study.

### Maternal outcome

The main clinical and analytical variables of the study participants are presented in table 1.

**Table 1:** Clinical, metabolic and ultrasound characteristics of the population studied.

	NGT (129)	GDM (78)	*P*
**Maternal characteristics**			
Age (years)	31.36±4.92	31.81±5.23	.533
Gestational age (weeks)	27 (26–28.75)	28 (27–29)	.229
Pre-pregnancy BMI (kg/m^2^)	23.12 (21.23–27.67)	24.72 (22.34–28.36)	.075
Gain in BMI (kg/m^2^)	4.83±2.05	3.70±2.06	**<.001**
SBP (mm Hg)	116.05±13.86	118.68±12.56	.172
DBP (mm Hg)	68.09±9.73	68.0±8.77	.993
Tobacco use n (%)	20 (15.50)	16 (20.51)	.736
Insulin treated n (%)	-	29 (37.66)	-
Fasting glucose (mg/dL)	80.34±6.89	85.78±10.37	**<.001**
Fasting insulin (µIU/mL)	7.46 (5.82–12.85)	10.05 (7.11–15.19)	**.005**
HOMA-IR	1.49 (1.11–2.52)	2.17 (1.44–3.56)	**.001**
Cholesterol (mmol/L)	6.68±1.10	6.62±1.10	.699
HDL cholesterol (mmol/L)	1.92±0.32	1.85±0.33	.187
Triglycerides (mmol/L)	1.98±0.62	2.19±0.73	**.029**
Activin A (ng/mL)	1.75 (1.34–2.35)	1.78 (1.30–2.54)	.499
Follistatin (ng/mL)	9.22±3.41	8.21±2.32	**.012**
FSTL3 (ng/mL)	12.45±4.06	12.01±3.39	.418
**Neonatal characteristics**			
Gestational age at delivery	39 (38–40)	39 (38–40)	.560
Male sex n (%)	60 (46.5)	41 (52.6)	.400
Birth weight (g)	3276.90±479.21	3246.60±461.92	.655
Birth weight (SDS)	0.12±1.08	0.13±1.07	.926
Percent fat mass (n) (%)	(96) 9.84±1.67	(69) 9.57±1.54	.285
FWE_28_ SDS (n)	(120) 1.12±0.90	(77) 1.12±0.76	.999
FWE_35_ SDS (n)	(125) 0.88±0.90	(77) 0.77±0.89	.398
FTVE_28_ SDS (n)	(103) 0.12±3.14	(68) 0.35±4.27	.693
FTVE_35_ SDS (n)	(103) −1.22±6.82	(70) −1.30±7.45	.942

Value data are presented as mean ± SD or median (25^th^–75^th^ percentile) for non-normally distributed variables. SBP: systolic blood pressure, DBP: diastolic blood pressure, FWE: fetal weight estimation, FTVE: fractional thigh volume estimation SDS: standard deviation score.

**Figure 1:**
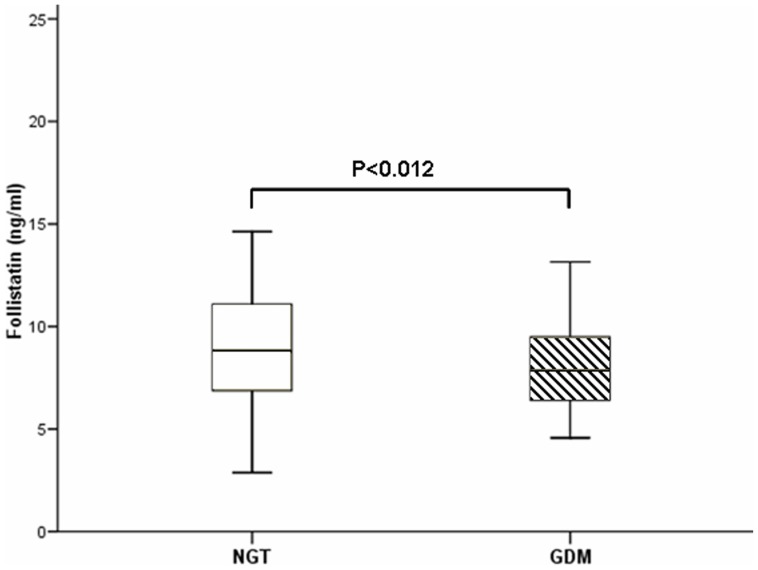
Serum follistatin levels in the NGT group compared with the GDM group.

As expected, fasting glucose and insulin levels, HOMA-IR index and triglycerides levels were higher in GDM than in NGT women. By contrast NGT women showed a higher gain in BMI during pregnancy whereas pre-pregnancy BMI was similar in both groups. Serum follistatin levels were significantly lower in the GDM group compared with the NGT group (9.22±3.41 vs. 8.21±2.32 ng/ml, *P* = 0.012) (Figure 1), whereas serum activin A and FSTL3 concentrations were comparable between the two groups.

### Fetal outcome

The main clinical and analytical variables of the offspring are summarized in table 1.

Gender distribution, mean BW SDS, PFM and gestational age at delivery were similar in both groups. There were no differences in FTVE_28_ SDS and FTVE_35_ SDS between the two groups. Also, FWE_28_ SDS and FWE_35_ SDS were comparable in the two groups.

### Bivariate correlation analysis

#### Relationship between activin A and clinical, analytical and ultrasound variables

Circulating activin A levels were weakly correlated with serum triglycerides (*r* = 0.185; *P* = 0.008) and maternal age (*r* = 0.142; *P* = 0.042). Also, a positive relationship was observed with FWE_28_ SDS (*r* = 0.183, *P* = 0.034).

When the analysis was performed separately in the NGT group the association with triglycerides disappeared, but the correlation with maternal age remained significant (*r* = 0.185; *P* = 0.036).

In contrast, Activin A levels in the GDM group were unrelated with any of the clinical, analytical or ultrasound parameters.

#### Relationship between follistatin and clinical, analytical and ultrasound variables

In the whole group, serum follistatin levels were negatively correlated with maternal age (*r* = −0.167; *P* = 0.016). Regarding metabolic variables, a negative association was found with fasting glucose (*r* = −0.176; *P* = 0.011), fasting insulin (*r* = −0.141; *P* = 0.042) and HOMA-IR index (*r* = −0.153; *P* = 0.030). Likewise, a positive correlation was observed with ultrasound parameters, FTVE_35_ SDS (*r* = 0.212; *P* = 0.005) and FWE_35_ SDS (*r* = 0.142; *P* = 0.014), and with anthropometric neonatal parameters, BW SDS (*r* = 0.152; *P* = 0.029) and PFM (*r* = 0.239; *P* = 0.002).

When the analysis was performed in the GDM group, only maternal age was negatively associated with serum follistatin concentrations (*r* = −0.275; *P* = 0.015) and the positive correlations with ultrasound parameters were lost. On the other hand, in the NGT group, the correlations observed with BW SDS and PFM remained significant and stronger (*r* = 0.215; *P* = 0.015 and *r* = 0.307; *P* = 0.002, respectively). Also, FTVE_35_ SDS and FTVE_28_ SDS were positively associated with serum follistatin levels (*r* = 0.249; *P* = 0.011 and *r* = 0.202; *P* = 0.042, respectively).

#### Relationship between FSTL3 and clinical, analytical and ultrasound variables

Circulating FSTL3 levels were only positively correlated with activin A (r = 0.308, P = 0.001) in the whole group. This correlation persists in the NGT group (*r* = 0.265, *P* = 0.002) and in the GDM group (*r* = 0.403, *P* = 0.001).

### Multivariate regression analysis

To assess the main independent determinants of activin A, follistatin and FSLT3 levels different stepwise multiple linear regression analyses were constructed. In each model, activin A, follistatin and FSLT3 were introduced as dependent variables. Maternal age, diagnosis of GDM, pre-pregnancy BMI, BMI gain, HOMA-IR index and triglycerides levels were introduced as variables for selection.

Activin A levels were positively associated with triglycerides concentrations and maternal age and negatively with pre-pregnancy BMI. When we also introduced follistatin and FSTL3 for selection, FSTL3 and triglycerides remained the sole positive determinants of activin A concentrations, explaining 13% of its variability (table 2)

**Table 2:** Stepwise multiple linear regression models of activin A, follistatin and FSTL3.

Dependent Variable	Covariates	Standardized Beta	*P*
LogActivinA (*r^2^* = 0.078; *P* = .001)*	Triglycerides	0.217	.002
	Maternal age	0.160	.021
	LogPre-pregnancy BMI	−0.142	.044
LogActivinA (*r^2^* = 0.131; *P*<.001)**	FSTL3	0.304	<.001
	Triglycerides	0.177	.008
Follistatin (*r^2^* = 0.099; *P*<.001)	Maternal age	−0.186	.007
	LogHOMA-IR	−0.199	.008
	Triglycerides	0.177	.016
	Diagnosis of GDM	−0.138	.049
FSLT3 (*r^2^* = 0.099; *P*<.001)	LogActivin A	0.315	<.001

* Model 1: Covariates considered for selection: maternal age, diagnosis of GDM, LogHOMA-IR, LogPre-pregnancy BMI, BMI gain, and triglycerides.

**Model 2: Covariates considered for selection: The same as in model 1, follistatin and FSTL3.

*r^2^*: corrected R^2^.

Regarding follistatin determinants, circulating levels were negatively associated with maternal age, HOMA-IR index and the diagnosis of gestational diabetes, and positively with triglycerides concentrations. The model explained approximately 10% of follistatin variability, and the inclusion of activin A or FSTL3 did not improve the percentage explained by the model (*r* ^2^ = 0.099) (table 2)

FSTL3 was unrelated with any of the clinical or metabolic variables. When we introduced activin A for selection, this variable was included in the model and explained approximately 10% of the variability of FSTL3 levels (*r* ^2^ = 0.099) (table 2)

We also analyzed which variables were associated with birth weight, percent fat mass, fetal weight and fractional thigh volume estimated by ultrasound examination. Each one of these measures was introduced as a dependent variable and the variables for selection were activin A, follistatin, FSTL3 and all the previous maternal clinical and analytical variables.

Follistatin levels in conjunction with BMI gain and maternal pre-pregnancy BMI were positive determinants of BW SDS, PFM and FWE_35_ SDS, whereas FTVE_35_ SDS was exclusively related with follistatin concentrations (table 3).

**Table 3:** Stepwise multiple linear regression models of anthropometrical and ultrasound variables.

Dependent Variables	Covariates	Standardized Beta	*P*
BW SDS (*r^2^* = 0.101; *P*<.001)	BMI gain	0.237	.001
	LogPre-pregnancy BMI	0.250	.001
	Follistatin	0.148	.031
PFM (*r^2^* = 0.159; P<.001)	BMI gain	0.317	<.001
	Follistatin	0.231	.002
	LogPre-pregnancy BMI	0.245	.002
FTVE_35_ SDS (*r^2^* = 0.046; *P* = .005)	Follistatin	0.214	.005
FWE_35_ SDS (r^2^ = 0.073; *P* = 0.002)	LogPre-pregnancy BMI	0.224	.003
	BMI gain	0.161	.030
	Follistatin	*0.149*	.034

Covariates considered for selection: LogActivin A, follistatin, FSTL3, maternal age, diagnosis of GDM, LogHOMA-IR, LogPre-pregnancy BMI, BMI gain and triglycerides. BW: birth weight, SDS: standard deviation score, PFM: percent fat mass, FTVE_35_: Fractional thigh volume estimation calculated at approximately 35 weeks' gestation, FWE_35_: fetal weight estimation calculated at approximately 35 weeks' gestation, *r^2^*: corrected R^2^.

## Discussion

In this study, we report for the first time that serum follistatin concentrations are lower in GDM than in NGT pregnant women, although the effect size measure was 2.5%. No differences in FSTL3 and activin A serum levels were observed between the two groups. Follistatin and FSTL3 share some structural and functional homologies including inhibition of activin A bioactivity *in vivo*. They also bind other members of the TGF-beta superfamily such as myostatin with important implications in muscle development and glucose homeostasis [30]. In this line, FSTL-3 has been recently explored in the setting of pregnancy as a potential predictive biomarker for GDM with divergent results [19], [20]. When this protein has been explored at the end of pregnancy, low circulating levels in parallel with a low expression in placental tissue in GDM patients have been described in a Chinese population [21]. However, our data do not support a major role of FSTL3 either in the metabolic profile observed in GDM women or in neonate anthropometric outcomes. In fact, in our cohort follistatin was the only studied protein that was weakly negatively associated with the presence of GDM and with HOMA-IR index. These data are in accordance with a recent study that reports reduced levels of circulating follistatin levels in patients with type 2 diabetes [31]. We are aware that the observational design of our study does not permit to infer mechanistic conclusions. However after controlling for other confounding factors the diagnosis of gestational diabetes remains a negative determinant of serum follistatin concentrations, leading us to suggest a possible role of follistatin in the carbohydrate metabolism during pregnancy.

Despite both follistatin and FSTL3 proteins displaying activin A inhibitory activity, they also have functional biological differences, mainly attributed to cell-surface binding activity and ligand specificity [10]. In fact, a very recent report determines that the N-terminal domain of follistatin molecules is critical for fitting ligand antagonists [32]. Thus, we believe that both molecules should be considered together to better interpret circulating levels when studying a specific condition. This may be one of the reasons for the inconsistent results observed for FSTL3 serum levels in the context of GDM-complicated pregnancy.

There are few data regarding activin A levels in the context of GDM-complicated pregnancy. Increased levels of activin A in GDM patients has been reported in previous studies [17], [18] with a decrease to normal range after starting insulin therapy for diabetes treatment. Circulating activin A concentrations have also been shown to be increased in preeclampsia and fetal growth restriction [33], but not in small-for-gestational-age newborns [5]. We have found no differences in serum activin A levels regarding carbohydrate metabolism. In our cohort, FSLT-3 and triglyceride levels were the sole determinants of circulating activin A levels. These apparent discrepancies may account for several aspects, ranging from the type of method for analyzing activin A in serum samples to the number of pregnant women recruited for our study, which is quite large in the present cohort compared to previous studies. Activin A has also been proposed to have a pro-inflammatory effect, stimulating the secretion of TNF alpha and IL6 cytokines [34], both well-known important players in the pathogenesis of insulin resistance. Despite not addressing this point in our study, the absence of association with HOMA-IR index in our patients and the similar circulating levels of this glycoprotein in healthy and GDM groups makes it unlikely for the proposed inflammatory effect of this molecule to be relevant in insulin resistance during pregnancy and in the carbohydrate metabolic defect observed in GDM patients.

An important role of activin A and follistatin has recently been described in the control of adipogenesis. Several studies identify these molecules as new adipokines expressed in adipose tissue with potential effects on obesity [9], [11]. Adipokines arise as important determinants of maternal metabolic homeostasis during pregnancy. Low expression levels of follistatin in adipose tissue from obese women have been linked with insulin resistance and hypertrophic obesity traits [11]. Interestingly, in our cohort maternal circulating follistatin levels were positively associated in the whole group and in the NGT group, with several markers of fetal and neonatal adiposity although these correlations are lost in the GDM group. Despite no clear explanation for the different behavior observed between the two groups, we think that therapeutic intervention introduced in the GDM group may play a role. It is well known, that offspring of GDM-treated women normalize neonatal weight, which entails a change in fetal growth velocity and adipose tissue accretion during the third trimester. As follistatin levels were obtained before introducing therapy, the expected relationship may be altered by this intervention. In the multivariate analysis circulating follistatin concentrations emerged as positive determinants of FWE_35_ SDS, FTVE_35_ SDS, BW SDS and PFM at birth, independent of such well-established markers such as BMI gain and pre-pregnancy BMI. In our study corrected R2 shows low values, therefore these models are not valid for predictive purposes, and only weak associations can be inferred between the variables. However, these results open a new perspective regarding the control of fetal adiposity and body weight besides glucose metabolism during pregnancy, highlighting the role of the TGF-β system.

The strengths of our study include prospective data collection in a large cohort of pregnant women, concurrent testing of activin A, follistatin and FSTL3 in the early third trimester and a correlation of maternal serum data with ultrasound estimations and neonatal anthropometry of their offspring. This study also has some limitations. As we remark above, it is not possible to establish causality with a cross-sectional study design. Moreover, we have only included Caucasian women and so we cannot extrapolate these data to other ethnic groups. In addition, fetal adiposity has been estimated by fractional limb volume which is considered a surrogate measure.

In conclusion, we report that circulating follistatin levels are reduced in GDM compared with NGT pregnant women suggesting a possible role in glucose homeostasis during pregnancy. We also describe that maternal follistatin levels are independent positive determinants of fetal growth and neonatal adiposity suggesting that follistatin may be involved in the regulation of energy metabolism during fetal life. Further studies are needed to discern the exact role of the Activin A-Follistatin system on fetal growth and glucose metabolism in pregnancy.
